# The Small GTPase Rsg1 is important for the cytoplasmic localization and axonemal dynamics of intraflagellar transport proteins

**DOI:** 10.1186/2046-2530-2-13

**Published:** 2013-10-07

**Authors:** Eric R Brooks, John B Wallingford

**Affiliations:** 1 Section of Molecular Cell and Developmental Biology and the Institute for Cellular and Molecular Biology, The University of Texas at Austin, Patterson Labs, 2401 Speedway, Austin, TX 78712, USA; 2 The Howard Hughes Medical Institute, Chevy Chase, USA

**Keywords:** Cilia, Fuz, IFT, PCP, Rsg1

## Abstract

**Background:**

Cilia are small, microtubule-based protrusions important for development and homeostasis. We recently demonstrated that the planar cell polarity effector protein Fuz is a critical regulator of axonemal intraflagellar transport dynamics and localization. Here, we report our findings on the role of the small GTPase Rsg1, a known binding partner of Fuz, and its role in the dynamics and cytoplasmic localization of intraflagellar transport proteins.

**Results:**

We find that Rsg1 loss of function leads to impaired axonemal IFT dynamics in multiciliated cells. We further show that Rsg1 is required for appropriate cytoplasmic localization of the retrograde IFT-A protein IFT43. Finally, we show that Rsg1 governs the apical localization of basal bodies, the anchoring structures of cilia.

**Conclusions:**

Our data suggest that Rsg1 is a regulator of multiple aspects of ciliogenesis, including apical trafficking of basal bodies and the localization and dynamics intraflagellar transport proteins.

## Background

Cilia are small cellular organelles found across the eukaryotic lineage; they are composed of an axoneme, formed by membrane enclosed microtubule doublets growing from the basal body, an anchoring and nucleating structure [1]. In most organisms, from the green algae *Chlamydomonas reinhardtii* to humans, cilia are built and maintained by the highly conserved intraflagellar transport (IFT) system [1-3]. The IFT system is composed of ~20 proteins divided into two biochemically and genetically distinct sub-complexes, IFT-B, which governs anterograde traffic from the base of the cilia to the distal tip, and IFT-A, which governs the retrograde return to the cell body. These two sub-complexes assemble and then multimerize into complexes known as IFT trains, which attach to microtubule motors and undergo a transport cycle through the cilium [1,3,4]. Mutations in IFT-B proteins often lead to a complete loss of the cilium, whereas IFT-A mutations often lead to short cilia with abnormal accumulations of IFT-B at the distal tip. In either case, ciliary structure and function are severely compromised (see [1] for a recent review).

In vertebrates, most cells possess a single non-motile cilium which functions as a signal transduction center, most notably for Sonic Hedgehog signals critical for development [3,5,6]. However, specialized multiciliated cells (MCCs) contain dozens of motile cilia, which beat in a polarized and coordinated fashion to drive directed fluid flow across epithelia. Such cells are found, for example, in the mammalian airway, in the ventricles and spinal cord of the central nervous system, and in the oviduct. Therefore, dysfunction of multi-ciliated cells leads to defects in respiration, axon guidance, and reproduction [7-9].

Recent reports from several labs have suggested that the MCCs of the embryonic *Xenopus* epidermis are an excellent model system for the study of basic MCC differentiation and behavior [10-15]. We recently developed techniques to investigate the localization and dynamics of IFT proteins in *Xenopus* MCCs. Using these tools, we demonstrated that the planar cell polarity (PCP) protein Fuz, which we previously showed to be required for ciliogenesis [14,16], is important for localizing at least one IFT-A member to basal bodies. As a result, there is a failure to incorporate the retrograde machinery into axonemal IFT trains leading to a failure of retrograde trafficking and a resultant failure to maintain the axoneme [17].

Our lab has also shown that Fuz binds the putative small GTPase, Rsg1, and that loss of Rsg1 results in ciliogenesis phenotypes. Given the defective cytoplasmic localization of IFT upon loss of Fuz and the strong role for some GTPases in ciliogenesis and cytoplasmic trafficking [18-20], whether Rsg1 also played a role in the localization and dynamics of IFT was investigated. Herein, knockdown (KD) of Rsg1 function is shown to lead to similar, but non-identical defects in axonemal IFT dynamics as compared to loss of Fuz. Rsg1 KD is also shown to lead to cytoplasmic IFT organization defects similar to those observed upon Fuz perturbation and to a disorganization of apically localized basal bodies, a phenotype not observed in Fuz KD conditions. Together, these results suggest that Fuz and Rsg1 play similar, but not completely overlapping functions in ciliogenesis. They also suggest that Rsg1 may play a role in multiple aspects of ciliogenesis.

## Methods

### Embryo manipulations

Female adult *Xenopus laevis* were ovulated by injection of human chorionic gonadotropin and eggs were fertilized *in vitro,* dejellied in 3% cysteine (pH 7.9), and subsequently reared in 0.3? Marc?s Modified Ringer?s (MMR) solution. For microinjections, embryos were placed in a solution of 2.5% Ficoll in 0.3? MMR, injected using forceps and an Oxford universal micromanipulator, reared in 2.5% Ficoll in 0.3? MMR to stage 9, and then washed and reared in 0.3? MMR alone. Embryo culture, solutions and *in vitro* transcription were performed using standard protocols [21]. The University of Texas at Austin Institutional Animal Care and Use Committee monitored ethical animal use under protocol number AUP-2012-00156.

### Plasmids and cloning

GFP-IFT20, GFP-IFT43, RFP-CLAMP, GFP-MAP7, membrane-RFP, and centrin-RFP were all used as previously described [17].

### Morpholino and mRNA injections

Capped mRNA was synthesized using mMessage mMachine kits (Ambion). The translation blocking Rsg1 morpholino (5?-GGCCCGTATCTCTGT-3?) has been previously described [16]. We obtained a second, non-overlapping translation-blocking morpholino against Rsg1, termed Rsg1 KD2 (5?AGCTTCCGGTAACAAGTCAGTGCAG-3?). mRNAs and/or morpholinos were injected into two ventral blastomeres at the four cell stage to target the embryonic epidermis. mRNAs were injected at 50?200 pg per blastomere and both morpholinos were injected at 35 ng per blastomere.

### IFT imaging

High-speed *in vivo* imaging of IFT has been previously described [17]. Briefly, stage 26/27 *Xenopus* embryos [22] expressing the IFT fusion construct of interest were mounted flank down in 0.8% low-melting-point agarose in 0.3x MMR, as described by Kieserman et al. [23]. Time-lapse confocal series were captured with an LSM 5LIVE inverted microscope (Carl Zeiss) with a Plan NeoFluar 100?/1.3 oil immersion objective (Carl Zeiss). For axoneme compartment and basal body imaging, embryos were mounted as above and imaged on an inverted LSM PASCAL confocal microscope (Carl Zeiss) with a Fluar 100?/1.3 oil immersion objective (Carl Zeiss). For axonemal IFT intensity imaging, embryos were mounted as above and imaged with a LSM 700 (Carl Zeiss) using a Plan-APOCHROMAT 63?/1.4 oil immersion objective (Carl Zeiss).

### Image analysis and quantification

Axonemal compartment lengths were measured using hand-drawn lines in the Fiji distribution of ImageJ (NIH). Axonemal IFT intensities were measured by using a hand drawn line in Fiji to quantify the mean intensity of IFT along the length of an axoneme and dividing this value by the mean intensity of the membrane-RFP along the same line. Basal body foci and their associated IFT pools were detected using the Fiji 3D object counter plug-in. Object size was set to 20 and threshold was determined empirically to maximize detection of apparent foci. Various quantitative measures of these foci were then used as described in the text. All data were plotted using Prism 5 (GraphPad Software) and statistical comparisons were made by use of the Mann?Whitney *U* test in this software. The threshold for significance was set at *P* = 0.01. Figure images were processed for clarity in Imaris (Bitplane) and Photoshop (Adobe). All enhancements were applied uniformly to the whole image.

## Results and Discussion

Little is known about how the axoneme is patterned along its proximodistal axis. Others have recently demonstrated that specific dynein isoforms are distributed in a restricted fashion along this axis in motile respiratory cilia and that this pattern is functionally important [24,25]. In addition, a recent report has suggested that the proximodistal pattern in primary cilia is important for modulating Sonic Hedgehog signaling [26]. We previously showed that the microtubule binding protein CLAMP tagged with RFP is enriched in a specific distal axoneme compartment (~2 ?m) and also weakly decorates the entire proximal axoneme (Figure?1a?). Further, we demonstrated that this distal compartment was compromised upon Fuz KD [17]. We also demonstrated that proximal ciliary identity, marked by a construct consisting of GFP fused to the microtubule-binding domain of MAP7 (GFP-MAP7), was only minimally perturbed in Fuz KD axonemes.

**Figure 1:**
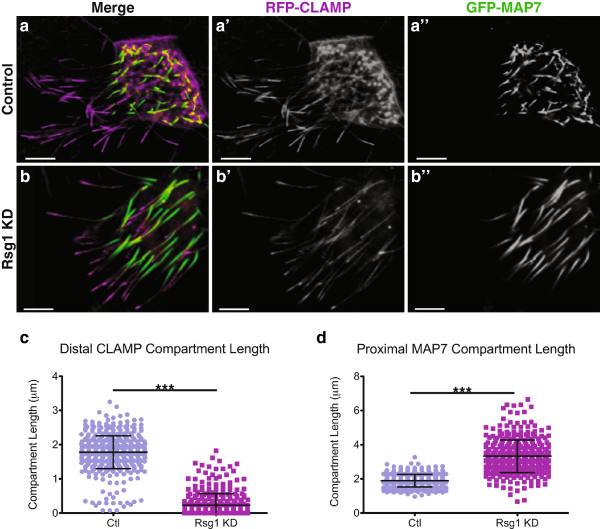
**Control of axonemal proximodistal patterning by the small GTPase Rsg1. (a?a?)** A representative *Xenopus* multiciliated cell (MCC), co-expressing GFP-MAP7, a marker of proximal ciliary identity, and RFP-CLAMP, a marker of the distal-tips of cilia. **(b?b?)** A MCC co-expressing GFP-MAP7 and RFP-CLAMP, and in which the function of the small GTPase, Rsg1, has been knocked-down (KD) by a translation-blocking antisense morpholino oligonucleotide. Note the significantly shortened or absent distal compartments of RFP-CLAMP as compared to controls. In addition, the proximal compartment marked by GFP-MAP7 is significantly expanded in these axonemes. This cell exhibits a moderate Rsg1 KD phenotype, and was chosen to facilitate direct comparison with the control cell. **(c)** Quantification of axonemal RFP-CLAMP compartments reveals a severe reduction in distal identity upon Rsg1 KD (Ctl [mean ? SD]: 1.78 ? 0.48 ?m, n = 517 axonemes, 29 cells, 5 embryos vs. Rsg1 KD: 0.23 ? 0.34 ?m, n = 361 axonemes, 28 cells, 5 embryos; ***P <0.0001). **(d)** Quantification of GFP-MAP7-positive compartments reveals a significant increase in proximal identity (Ctl: 1.90 ? 0.36 ?m, n = 452 axonemes, 29 cells, 5 embryos vs. Rsg1 KD: 3.32 ? 0.95 ?m, n = 364 axonemes, 39 cells, 5 embryos; ***P <0.0001). Scale bars represent 5 ?m.

To begin exploring the role of Rsg1 in ciliogenesis, we analyzed proximodistal axoneme patterning by Rsg1 KD with a previously validated antisense morpholino oligonucleotide [16] and analysis of the distribution of RFP-CLAMP and GFP-MAP7. Generally, axonemes were shorter in Rsg1 KD cells, and we found a severe reduction in the length of the CLAMP-positive distal compartment in Rsg1 KD cilia (Figure?1a?,b?,c), a more severe variant of the phenotype we observed in Fuz KD MCCs [17]. Surprisingly, however, we found a significant increase in MAP7-positive proximal ciliary identity upon Rsg1 KD, a phenotype not observed in Fuz KD cilia (Figure?1a?,b?,d; [17]). This result suggests that Rsg1 may have functions independent of Fuz, or that their functional relationship is not simply one-to-one.

This difference in axonemal proximodistal patterning led us to apply high-speed confocal IFT imaging approaches to MCCs lacking Rsg1 function. High-speed imaging of axonemes from control cells expressing GFP-IFT20, an anterograde IFT-B member [27], showed highly dynamic and processive bi-directional IFT trains, as previously observed (Figure?2a; Additional file 1: Movie 1; [17]). However, axonemes from Rsg1 KD MCCs showed disruption of these dynamics (Figure?2b; Additional file 2: Movie 2). We were unable to reliably follow IFT trains in most axonemes, as the entire length of the axoneme seemed to be filled with a low level of GFP-IFT20 (Figure?2b? vs. Figure?2a?). While we did observe some dynamic, train-like movement, we were unable to effectively track and quantify this behavior due to the increased background. One possible explanation for this is that the trains are misformed, either smaller or containing fewer IFT sub-complexes, leading to a reduced signal.

**Figure 2:**
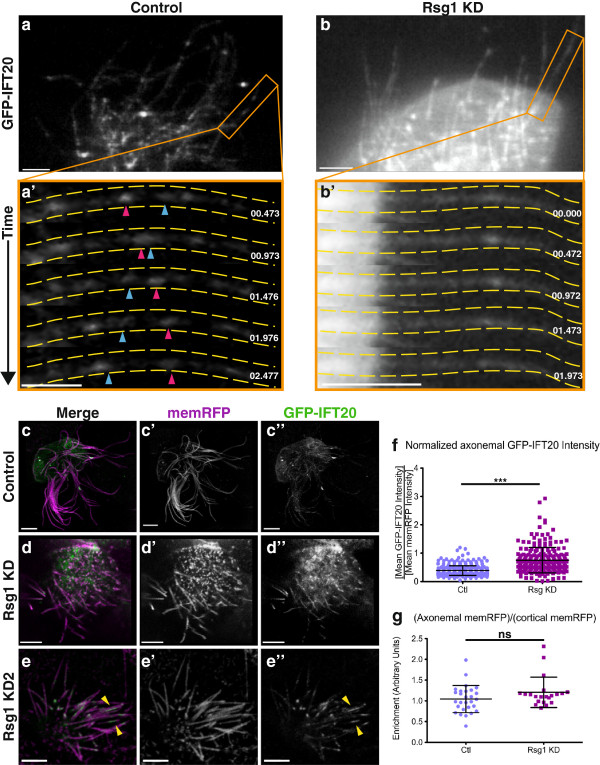
**GFP-IFT20 accumulates in axonemes of Rsg1 KD MCCs. (a)** High-magnification confocal image of axonemes from a control MCC expressing GFP-IFT20. The orange box indicates the region shown in **a?**. See also Additional file 1: Movie 1. **(a?)** A series of stills from Additional file 1: Movie 1. Yellow dashed lines outline the axoneme and distal is to the right. Pink and blue arrowheads indicate an anterograde train and a retrograde train, respectively. **(b)** High-magnification confocal image of axonemes from an Rsg1 KD MCC expressing GFP-IFT20. Orange box represents the region shown in **b?**. See also Additional file 2: Movie 2. **(b?)** A series of stills from Additional file 2: Movie 2. Yellow dashed lines outline the axoneme and distal is to the right. No IFT trains are visible during the course of the movie. **(c?c?)** A single confocal slice of an control MCC co-expressing membraneRFP and GFP-IFT20. **(d?d?)**. A single confocal slice of an Rsg1 KD MCC co-expressing membraneRFP and GFP-IFT20. **(e?e?)** A single confocal slice of an Rsg1 KD2 (second-site morpholino) MCC co-expressing membrane-RFP and GFP-IFT20. Yellow arrowheads indicate large GFP-IFT20 accumulations in distal axonemes. **(f)** Quantification of mean GFP-IFT20 signal along control and Rsg1 KD axonemes, as normalized to membrane-RFP intensity along the same length. Note the increase in normalized IFT20 signal in Rsg1 KD axonemes (Ctl: 0.39 ? 0.17, n = 304 axonemes, 36 cells, 7 embryos vs. Rsg1 KD: 0.75 ? 0.45, n = 223 axonemes, 31 cells, 6 embryos; ***P <0.0001). **(g)** There is no significant difference in axonemal average membrane-RFP signal as normalized to cortical membrane-RFP signal from the same cell, between control and Rsg1 KD conditions (Ctl: 1.04 ? 0.33, n = 28 cells, 6 embryos vs. Rsg1 KD: 1.21 ? 0.36, n = 21 cells, 6 embryos; *P* = 0.2607). Scale bars in **a?b?** indicate 3 ?m. Scale bars in **c-e?** indicate 5 ?m. Time stamps in **a?** and **b?** are relative to the first frame of Additional file 1: Movie 1 and Additional file 2: Movie 2, respectively.

We hypothesized that the high GFP-IFT20 background levels observed in Rsg1 KD axonemes might represent a more diffuse form of the IFT accumulation phenotype observed in Fuz KD MCCs [17]. To test this hypothesis, single confocal slices of control and Rsg1 KD MCC ciliary tufts co-expressing GFP-IFT20 and membrane-RFP were taken. The mean intensity of GFP-IFT20 was normalized along the length of axonemes to the mean intensity of membrane-RFP along the same length, and this value was compared in control and Rsg1 KD conditions. This value was significantly increased on average in Rsg1 KD axonemes (Figure?2c,d,f), supporting an expanded accumulation of anterograde IFT in these cilia. It is possible that membrane-RFP localization to cilia itself was affected by Rsg1 KD. To test this possibility, axonemal RFP intensity was normalized against cortical membrane-RFP signal in MCCs. When this value was compared in control and Rsg1 KD MCCs, no significant differences were observed (Figure?2g). Additionally, to rule out MCC specific membrane-RFP trafficking defects, cortical MCC signal was normalized against the cortical signal of neighboring goblet cells. Again, no change in this value was observed between control and Rsg1 KD conditions (Ctl (mean ? SD): 1.13 ? 0.29, 15 cells, 6 embryos vs. Rsg1 KD: 1.06 ? 0.31, 19 cells, 6 embryos; *P* = 0.8082,). Together, these data suggest that the observed increase in normalized GFP-IFT20 signal is not due to a change in membrane-RFP localization.

To further control for the specificity of the previously validated morpholino [16], a second, completely non-overlapping morpholino (designated Rsg1 KD2) was used to confirm that these phenotypes were due to a specific loss of Rsg1 function. As expected, injection of Rsg1 KD2 led to shorter axonemes and to elevated IFT20 signal in axonemes as compared to controls (Figure?2e?e?, Ctl (mean ? SD): 0.23 ? 0.14, n = 200 axonemes, 30 cells, 3 embryos vs. Rsg1 KD2: 0.35 ? 0.16, n = 195 axonemes, 25 cells, 4 embryos; *P* <0.0001). In addition, a small number of cells injected with Rsg1 KD2 contained axonemes exhibiting large IFT20 accumulations, reminiscent of those observed upon Fuz KD (Figure?2e,e?; [17]).

These data suggest that Rsg1 is required for appropriate axonemal IFT dynamics, and Rsg1 KD phenotypes may be a variant of the IFT phenotypes previously observed in Fuz KD MCCs. Therefore, it is predicted that retrograde IFT axonemal localization would be reduced or absent upon Rsg1 KD. To test this prediction, high-speed confocal microscopy of MCCs expressing GFP-IFT43, an IFT-A protein associated with Sensenbrenner syndrome [28], was initially used. Control axonemes showed processive bi-directional trafficking, as expected (Figure?3a,a?; Additional file 3: Movie 3). However, axonemes from Rsg1 KD MCCs showed faint levels of GFP-IFT43, and dynamic trains could not be readily observed. In our previous study, Fuz KD led to a severe and obvious reduction of axonemal IFT43 levels, but IFT43 was still apparent in Rsg1 KD axonemes. To directly test IFT43 localization to axonemes, mean GFP-IFT43 levels were normalized to mean membrane-RFP levels as above. Rsg1 KD axonemes showed a significant decrease in average normalized IFT43 intensity (Figure?3c,d,f). The same analysis was performed after injection of the Rsg1 KD2 morpholino and similar results were obtained (Figure?3e?e?,g). Together, these data suggest that Rsg1 KD leads to a failure of IFT43 localization to axonemes, though to a lesser extent than Fuz KD [17].

**Figure 3:**
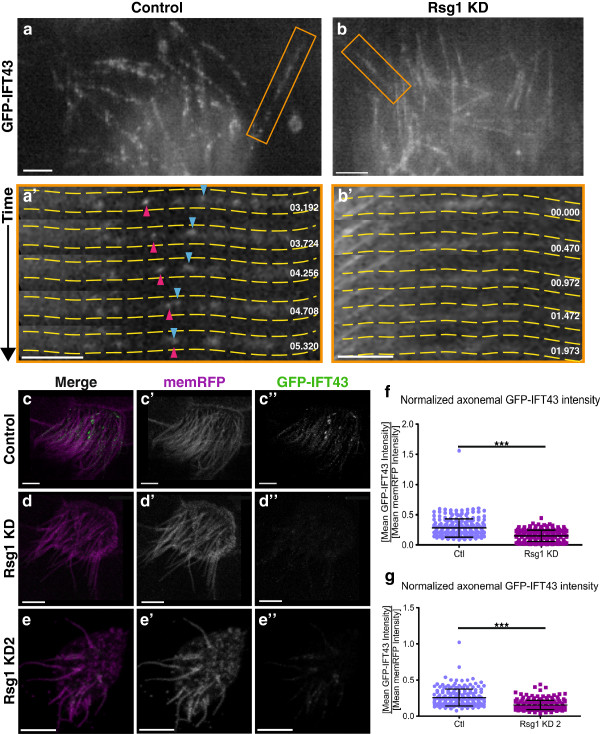
**GFP-IFT43 axonemal localization is reduced in Rsg1 KD MCCs. (a)** High-magnification confocal image of axonemes from a control MCC expressing GFP-IFT43. The orange box indicates the region shown in **a?**. See also Additional file 3: Movie 3. **(a?)** A series of stills from Additional file 3: Movie 3. Yellow dashed lines outline the axoneme, and distal is to the right. Pink and blue arrowheads indicate an anterograde train and a retrograde train, respectively. **(b)** High-magnification confocal image of axonemes from a Rsg1 KD MCC expressing GFP-IFT43. Orange box represents the region shown in **b?**. See also Additional file 4: Movie 4. **(b?)** A series of stills from Additional file 4: Movie 4. Yellow dashed lines outline the axoneme, and distal is to the right. No IFT trains are visible during the course of the movie. Note that the entire axoneme exhibits a faint uniform background signal. **(c?c?)** A single confocal slice of a control MCC co-expressing membrane-RFP and GFP-IFT43. **(d?d?)** A single confocal slice of an Rsg1 KD MCC co-expressing membrane-RFP and GFP-IFT43. **(e?e?)** A signal confocal slice of an Rsg1 KD2 MCC co-expressing membrane-RFP and GFP-IFT43. **(f)** Quantification of mean GFP-IFT43 signal along control and Rsg1 KD axonemes, as normalized to membrane-RFP intensity along the same length. Note the decrease in normalized IFT43 signal in Rsg1 KD axonemes (Ctl: 0.28 ? 0.15, n = 225 axonemes, 28 cells, 5 embryos vs. Rsg1 KD: 0.15 ? 0.09, n = 250 axonemes, 32 cells, 6 embryos; ***P <0.0001). **(g)** Quantification of mean GFP-IFT43 signal along control and Rsg1 KD2 axonemes, as normalized to membrane-RFP intensity along the same length (Ctl: 0.26 ? 0.12, n = 200 axonemes, 44 cells, 6 embryos vs. Rsg1 KD: 0.15 ? 0.06, n = 223 axonemes, 52 cells, 6 embryos; ***P <0.0001). Scale bars in **a?b?** indicate 3 ?m. Scale bars in **c?e?** indicate 5 ?m. Time stamps in **a?** and **b?** are relative to the first frame of Additional file 3: Movie 3 and Additional file 4: Movie 4, respectively.

In Fuz KD MCCs, IFT43, but not IFT20, fails to localize to cytoplasmic pools at basal bodies, and this is likely the cause of the axonemal IFT defects [17]. Given that Rsg1 KD axonemal phenotypes are so categorically similar to those of Fuz KD, we asked if the same failure to localize IFT43 to basal bodies also occurred in Rsg1 KD MCCs. To do this, single confocal slices of the apical surface of control and Rsg1 KD MCCs co-expressing either GFP-IFT20 or GFP-IFT43 along with the basal body marker centrin-RFP were taken (Figure?4a-f). Computational approaches were then used to quantify various properties of basal bodies and their associated IFT pools.

**Figure 4:**
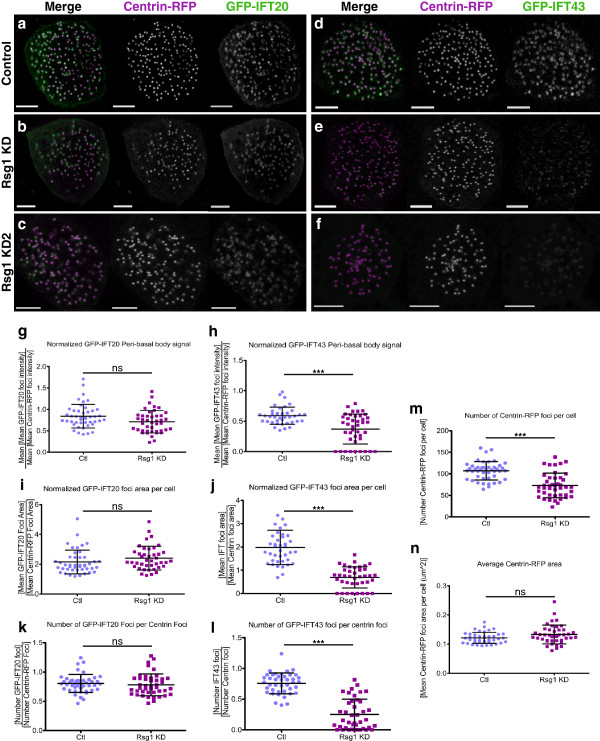
**GFP-IFT43 but not GFP-IFT20 requires Rsg1 function for localization to peri-basal body IFT pools. (a)** A single confocal slice of the apical surface of a control MCC expressing GFP-IFT20 and centrin-RFP. **(b)** A single confocal slice of a representative Rsg1 KD MCC co-expressing GFP-IFT20 and centrin-RFP. Note that despite the decreased density of centrin-RFP foci there is still a strong correlation between the centrin-RFP and GFP-IFT20 localization patterns. **(c)** A single confocal slice of a representative Rsg1 KD2 MCC co-expressing GFP-IFT20 and centrin-RFP. **(d)** A representative control MCC expressing GFP-IFT43 and centrin-RFP. **(e)** A representative Rsg1 KD MCC expressing GFP-IFT43 and centrin-RFP. Notice the impaired localization of GFP-IFT43 to centrin-RFP foci. **(f)** A representative Rsg1 KD2 MCC expressing GFP-IFT43 and centrin-RFP. **(g)** Quantification of the mean of GFP-IFT20 foci mean intensities, as normalized to the same value for centrin-RFP, shows no significant change between control and Rsg1 KD MCCs (Ctl: 0.84 ? 0.28, n = 45 cells, 8 embryos vs. Rsg1 KD: 0.71 ? 0.26, n = 43 cells, 8 embryos, *P* = 0.042). **(h)**. Quantification of the mean of GFP-IFT43 foci mean intensities, as normalized to the same value for centrin-RFP, shows a significant decrease between control and Rsg1 KD MCCs (Ctl: 0.59 ? 0.14, n = 41 cells, 8 embryos vs. Rsg1 KD: 0.37 ? 0.25, n = 41 cells, 8 embryos. ***P <0.0001). **(i)** Quantification of the mean area of GFP-IFT20 foci in a cell normalized against the same value for centrin-RFP shows no significant change between control and Rsg1 KD conditions (Ctl: 2.14 ? 0.79, n = 44 cells, 8 embryos vs. Rsg1 KD: 2.41 ? 0.80, n = 41 cells, 8 embryos; *P* = 0.3477). **(j)** Quantification of the mean area of GFP-IFT43 foci in a cell normalized against the same value for centrin-RFP shows a significant decrease in Rsg1 KD MCCs as compared to controls (Ctl: 1.98 ? 0.74, n = 39 cells, 8 embryos vs. Rsg1 KD: 0.69 ? 0.46, n = 39 cells, 8 embryos; ***P <0.0001). **(k)** There is no significant change in the number of GFP-IFT20 foci detected per centrin-RFP foci between control and Rsg1 KD MCCs (Ctl: 0.81 ? 0.15, n = 45 cells, 8 embryos vs. Rsg1 KD: 0.78 ? 0.19, n = 43 cells, 8 embryos; *P* = 0.062). **(l)** There is a significant reduction in the number of GFP-IFT43 foci detected per centrin-RFP foci between control and Rsg1 KD MCCs (Ctl: 0.76 ? 0.17, n = 41 cells, 8 embryos vs. Rsg1 KD: 0.25 ? 0.25, n = 41 cells, 8 embryos; ***P <0.0001). **(m)** There is a reduction in the number of centrin-RFP foci detected on average in Rsg1 KD MCCs as compared to controls (Ctl: 106.90 ? 21.39, n = 45 cells, 8 embryos vs. Rsg1 KD: 72.95 ? 28.63, n = 43 cells, 8 embryos; ***P <0.0001). **(n)** The average area of detected centrin-RFP foci is not significantly different between control and Rsg1 KD MCCs, indicating that there are no gross abnormalities in apically docked basal bodies upon Rsg1 KD (Ctl: 0.12 ? 0.02, n = 45 cells, 8 embryos vs. Rsg1 KD: 0.13 ? 0.03, n = 42 cells, 8 embryos; *P* = 0.685). Scale bars in **a?f** represent 5 ?m.

First, the mean of the mean intensities of all GFP-IFT20 foci in a cell were normalized against the same value for centrin-RFP. This value was statistically equivalent between control and Rsg1 KD MCCs, as expected from studies on Fuz (Figure?4g). Applying the same measure to GFP-IFT43 showed a modest, but significant decrease of GFP-IFT43 localization in Rsg1 KD conditions (Figure?4h). In addition, while some IFT43 foci were detectable by eye in Rsg1 KD MCCs (Figure?4e), they seemed smaller and misshapen as compared to the controls. To further explore this defect, the average size of detected GFP-IFT43 foci in a cell was normalized against the average size of centrin-RFP foci (which do not vary significantly between control and Rsg1 KD conditions; Figure?4n). As expected, this value was reduced in Rsg1 KD MCCs as compared to controls (Figure?4j). A similar analysis of GFP-IFT20 average foci area showed no significant difference between control and Rsg1 KD1 (Figure?4i). The number of detected IFT foci in a cell was compared to the number of detected centrin foci; no significant change was observed in the number of GFP-IFT20 foci per centrin-RFP foci between control and Rsg1 KD cells (Figure?4k). However, this value was significantly reduced in GFP-IFT43 MCCs (Figure?4l). These analyses were repeated for Rsg1 KD2 MCCs yielding similar results (Figure?4c,f; Additional file 5).

Finally, the apical array array seemed less densely populated in Rsg1 KD MCCs (Figure?4a-f). To test this, the number of centrin-RFP per MCC was quantified. While this value is variable even in control cells, it was found that on average, there were fewer centrin-RFP foci per cell between control and Rsg1 KD conditions (Figure?4m). However, the average absolute size of centrin-RFP foci was not significantly different upon Rsg1 KD, suggesting that detected basal bodies are likely appropriately formed (Figure?4n). These data could indicate that there is an apical trafficking defect of these basal bodies. To test this possibility, three-dimensional confocal stacks of single MCCs expressing centrin-RFP were taken and the distribution of centrin foci was analyzed. Control MCCs exhibit a stereotypical pattern whereby centrin-RFP foci are arrayed at the apical surface (Figure?5a). In Rsg1 KD MCCs, this pattern is variably perturbed, with either mild or severe disruption of the apical array, as well as clumps of centrin-RFP foci sitting below the apical surface (Figure?5b).

**Figure 5:**
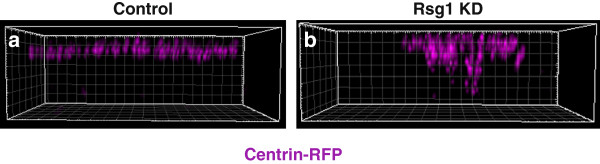
**Rsg1 controls apical trafficking of basal bodies. (a)** A 3D-reconstruction of the long axis of a control MCC shows a consistent localization of centrin-RFP foci to the apical surface. **(b)** A 3D-reconstruction of the long axis of an Rsg1 KD MCC shows a disorganization of centrin-RFP foci and failure of some foci to localize apically. The grid-boxes in **a** and **b** are in 1 ?m increments.

Interestingly, when the total number of centrin-RFP foci in 3D reconstructions of MCCs was quantified, a modest, though significant, decrease in the number of basal bodies was still observed upon Rsg1 KD (Ctl (mean ? SD): 147.4 ? 12.47 basal bodies, n = 21 cells, 4 embryos vs. Rsg1 KD: 124.6 ? 24.36 basal bodies, n = 21 cells, 6 embryos; *P* = 0.0012). There are at least two interpretations of this result. First, Rsg1 KD MCCs often have a large closely-knit clump of sub-apical centrin-RFP foci (Figure?5b), which makes accurate quantification difficult and could lead to the observed reduction. A second interpretation is that there is a small defect in basal body generation, which might also be consistent with the observed sub-apical mass of centrin foci; as in *Xenopus* MCCs, basal bodies are generated from sub-apical structures known as deuterostomes [10]. Thus, the observed mass of centrin foci could be centered on a Deuterosome undergoing defective basal body formation or failing to allow newly formed basal bodies to undergo appropriate trafficking.

Together, our data suggest that Rsg1 plays a role in the apical organization of basal bodies, an interesting finding, given that we did not observe this phenotype in Fuz KD MCCs [17]. Notably, however, three other PCP proteins, namely Dsh, Intu, and Celsr, do display apical basal body docking defects [15,29]. Therefore, Rsg1 may play a role in multiple PCP-dependent ciliogenic processes.

One question that remains is how, or even if, Fuz and Rsg1 are coupled in the process of IFT localization to basal bodies. One hypothesis is that one protein is reliant on the other for its localization and/or function. Several lines of evidence suggest that Rsg1 may be the regulator in this case; first, its nature as a putative GTPase already suggests a mechanism for regulation of binding and/or localization. Second, the basal body docking phenotype in Rsg1 KD MCCs suggests that Rsg1 may be playing a role in the earliest localization events of ciliogenesis, while Fuz acts only later. Third, while the ciliogenic phenotypes in Rsg1 KD MCCs appear more severe than those of Fuz KD MCCs (overall shorter axonemes and far sparser ciliary tufts, on average), axonemal IFT dynamics do not appear as perturbed as those in Fuz KD MCCs. One potential explanation for this is that Rsg1 controls the localization of a large number of ciliary proteins, including Fuz, while Fuz controls the localization of IFT43 and possibly other IFT-A proteins. Therefore, the partial loss of Rsg1 expected from incomplete knockdown would still allow some functional Fuz localization to basal bodies, which would allow for the formation of a small number of appropriately assembled IFT trains. As a result, IFT cycling dynamics might occur at a rate greater than allowed for by direct Fuz KD.

Future work will seek to clarify the relationship between Rsg1 and Fuz. One obvious question will be that of the dynamic localization of Fuz during ciliogenesis and its dependence on the function of Rsg1. More intriguing is the question of how Rsg1 function itself might be modulated, especially given its role in early ciliogenesis. Finally, it will be interesting to discover how Rsg1 interacts with the various other GTPases known to be involved in trafficking ciliogenic cargos [18,19,30].

## Conclusions

Our work demonstrates that the small GTPase Rsg1 is an important regulator of cytoplasmic IFT localization, similar to its binding partner Fuz [17]. In addition, we have demonstrated a role for Rsg1 in the apical organization of basal bodies, a role shared by other PCP proteins, but not by Fuz. Together, our data suggest that Rsg1 may be a multifunctional regulator of PCP-dependent ciliogenesis.

## Abbreviations

IFT: Intraflagellar transport; KD: Knockdown; MCCs: Multiciliated cells; PCP: Planar cell polarity.

## Competing interests

The authors declare that they have no competing interests.

## Author? contributions

EB and JW designed and interpreted all experiments. EB performed all experiments. EB wrote the manuscript. Both authors read and approved the final manuscript.

## Supplementary Material

Additional file 1 **Movie 1.** A high-speed confocal time-series of axonemes from a control MCC expressing GPF-IFT20. Note the highly processive, bi-directional traffic. Frames are taken every 0.5 seconds. Playback occurs at 5 frames per second. Scale bar represents 3 ?m. Click here for file

Additional file 2 **Movie 2.** A high-speed confocal time-series of axonemes from a Rsg1 KD MCC expressing GFP-IFT20. Note the decreased dynamics and the overall high background of the images. Frames are taken every 0.5 seconds. Playback occurs at 5 frames per second. Scale bar represents 3 ?m. Click here for file

Additional file 3 **Movie 3.** A high-speed confocal time-series of axonemes from a control MCC expressing GFP-IFT43. Note the highly processive, bi-directional traffic. Frames are taken every 0.5 seconds. Playback occurs at 5 frames per second. Scale bar represents 3 ?m. Click here for file

Additional file 4 **Movie 4.** A high-speed confocal time-series of axonemes from a Rsg1 KD MCC expressing GFP-IFT43. Note the decreased dynamics and rapid loss of signal in these images. Frames are taken every 0.5 seconds. Playback occurs at 5 frames per second. Scale bar represents 3 ?m. Click here for file

Additional file 5 **Quantification of centrin and IFT analyses from Rsg1 KD2 MCCs. (a)** Quantification of the mean of GFP-IFT20 foci mean intensities, as normalized to the same value for centrin-RFP, shows no significant change between control and Rsg1 KD2 MCCs (Ctl (mean ? SD): 0.57 ? 0.29, n = 48 cells, 7 embryos vs. Rsg1 KD2: 0.57 ? 0.33, n = 48 cells, 7 embryos; *P* = 0.8980). **(b)** Quantification of the mean of GFP-IFT43 foci mean intensities, as normalized to the same value for centrin-RFP, shows a significant decrease between control and Rsg1 KD MCCs (Ctl: 0.36 ? 0.09, n = 47 cells, 5 embryos vs. Rsg1 KD2: 0.21 ? 0.17, n = 46 cells, 5 embryos; ***P <0.0001). **(c)** Quantification of the mean area of GFP-IFT20 foci in a cell normalized against the same value for centrin-RFP shows no significant change between control and Rsg1 KD2 conditions (Ctl: 2.17 ? 0.62, n = 48 cells, 7 embryos vs. Rsg1 KD2: 2.51 ? 1.06, n = 48 cells, 7 embryos; *P* = 0.1212). **(d)** Quantification of the mean area of GFP-IFT43 foci in a cell normalized against the same value for centrin-RFP shows a significant decrease in Rsg1 KD2 MCCs as compared to controls (Ctl: 2.79 ? 0.86, n = 47 cells, 5 embryos vs. Rsg1 KD2: 1.50 ? 1.25, n = 46 cells, 5 embryos; ***P <0.0001). **(e)** There is no significant change in the number of GFP-IFT20 foci detected per centrin-RFP foci between control and Rsg1 KD2 MCCs (Ctl: 0.78 ? 0.13, n = 48 cells, 7 embryos vs. Rsg1 KD2: 0.78 ? 0.17, n = 48 cells, 7 embryos; *P* = 0.5504). **(f)** There is a significant reduction in the number of GFP-IFT43 foci detected per centrin-RFP foci between control and Rsg1 KD2 MCCs (Ctl: 0.78 ? 0.17, n = 47 cells, 5 embryos vs. Rsg1 KD2: 0.48 ? 0.32, n = 46 cells, 5 embryos; ***P <0.0001). Click here for file
