# Supplementary figures and images for: The Small GTPase Rsg1 is important for the cytoplasmic localization and axonemal dynamics of intraflagellar transport proteins
Source: Cilia. 2013 Oct 7;2:13. doi: 10.1186/2046-2530-2-13 (PMC3850895; doi:10.1186/2046-2530-2-13)

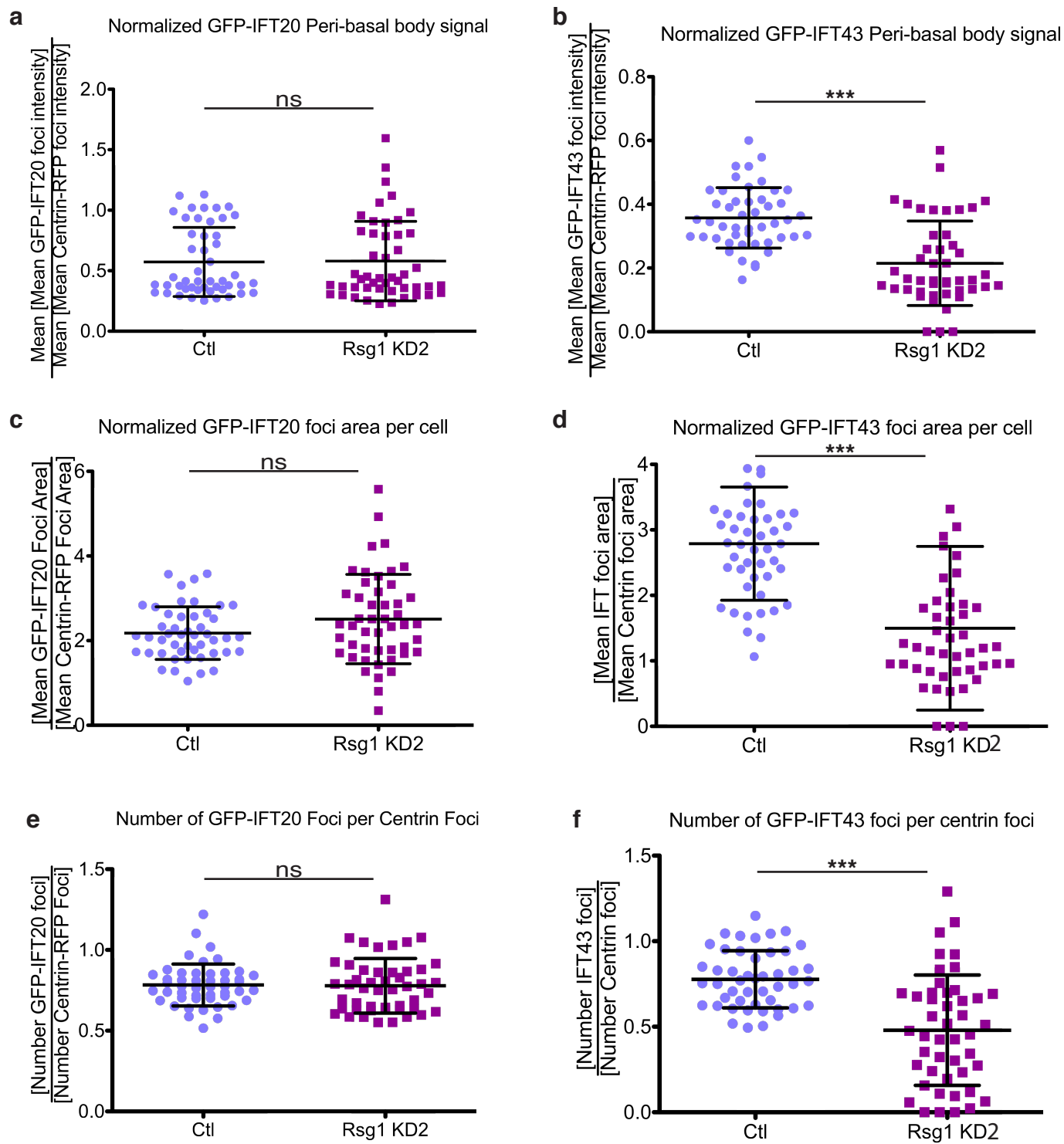

Supplement: Additional file 5 — Quantification of centrin and IFT analyses from Rsg1 KD2 MCCs. (a) Quantification of the mean of GFP-IFT20 foci mean intensities, as normalized to the same value for centrin-RFP, shows no significant change between control and Rsg1 KD2 MCCs (Ctl (mean ? SD): 0.57 ? 0.29, n = 48 cells, 7 embryos vs. Rsg1 KD2: 0.57 ? 0.33, n = 48 cells, 7 embryos; P = 0.8980). (b) Quantification of the mean of GFP-IFT43 foci mean intensities, as normalized to the same value for centrin-RFP, shows a significant decrease between control and Rsg1 KD MCCs (Ctl: 0.36 ? 0.09, n = 47 cells, 5 embryos vs. Rsg1 KD2: 0.21 ? 0.17, n = 46 cells, 5 embryos; ***P <0.0001). (c) Quantification of the mean area of GFP-IFT20 foci in a cell normalized against the same value for centrin-RFP shows no significant change between control and Rsg1 KD2 conditions (Ctl: 2.17 ? 0.62, n = 48 cells, 7 embryos vs. Rsg1 KD2: 2.51 ? 1.06, n = 48 cells, 7 embryos; P = 0.1212). (d) Quantification of the mean area of GFP-IFT43 foci in a cell normalized against the same value for centrin-RFP shows a significant decrease in Rsg1 KD2 MCCs as compared to controls (Ctl: 2.79 ? 0.86, n = 47 cells, 5 embryos vs. Rsg1 KD2: 1.50 ? 1.25, n = 46 cells, 5 embryos; ***P <0.0001). (e) There is no significant change in the number of GFP-IFT20 foci detected per centrin-RFP foci between control and Rsg1 KD2 MCCs (Ctl: 0.78 ? 0.13, n = 48 cells, 7 embryos vs. Rsg1 KD2: 0.78 ? 0.17, n = 48 cells, 7 embryos; P = 0.5504). (f) There is a significant reduction in the number of GFP-IFT43 foci detected per centrin-RFP foci between control and Rsg1 KD2 MCCs (Ctl: 0.78 ? 0.17, n = 47 cells, 5 embryos vs. Rsg1 KD2: 0.48 ? 0.32, n = 46 cells, 5 embryos; ***P <0.0001). [file 2046-2530-2-13-S5.pdf]
